# Rapid Preparation of Platinum Catalyst in Low-Temperature Molten Salt Using Microwave Method for Formic Acid Catalytic Oxidation Reaction

**DOI:** 10.3390/molecules29215128

**Published:** 2024-10-30

**Authors:** Haidong Zhao, Xiaoyan Hu, Hongbiao Ling, Ji Li, Weixu Wang, Jingtao Guo, Rui Liu, Chao Lv, Zhen Lu, Yong Guo

**Affiliations:** 1 School of Chemistry and Chemical Engineering, Shanxi Datong University, Datong 037009, China; zhaohd2004@126.com (H.Z.); 18602329176@163.com (X.H.); linghongbiao2024@163.com (H.L.); 03130045@sxdtdx.edu.cn (J.L.); wang_wx10@163.com (W.W.); 13509767724@163.com (J.G.); liurui198689@163.com (R.L.); 2 School of Coal Engineering, Shanxi Datong University, Datong 037009, China; lvchao0711@126.com

**Keywords:** fuel cell, Pt/C catalyst, formic acid, microwave method

## Abstract

In this paper, platinum nanoparticles with a size of less than 50 nm were rapidly and successfully synthesized in low-temperature molten salt using a microwave method. The morphology and structure of the product were characterized by SEM, TEM, EDX, XRD, etc. The TEM and SEM results showed that the prepared product was a nanostructure with concave and uniform size. The EDX result indicated that the product was pure Pt, and the XRD pattern showed that the diffraction peaks of the product were consistent with the standard spectrum of platinum. The obtained Pt/C nanoparticles exhibited remarkable electrochemical performance in a formic acid catalytic oxidation reaction (FAOR), with a peak mass current density of 502.00 mA·mg^−1^_Pt_ and primarily following the direct catalytic oxidation pathway. In addition, in the chronoamperometry test, after 24 h, the mass-specific activity value of the Pt concave NPs/C catalyst (10.91 mA·mg^−1^_Pt_) was approximately 4.5 times that of Pt/C (JM) (2.35 mA·mg^−1^_Pt_). The Pt/C NPs exhibited much higher formic acid catalytic activity and stability than commercial Pt/C. The microwave method can be extended to the preparation of platinum-based alloys as well as other catalysts.

## 1. Introduction

Recently, proton-exchange membrane fuel cells (PEMFCs) have attracted considerable attention in the field of fuel cells due to their high conversion efficiency and low cost [1,2,3]. Platinum (Pt) is the most widely used catalyst for PEMFCs, but its high cost, scarcity, and low utilization efficiency limit its commercial application [4]. Research into Pt-based catalysts has focused on controlling the morphology [5], reducing the nanoparticle (NP) size [6], doping Pt with non-noble metals [3], and developing single-atom catalysts (SACs) to maximize the efficacy of each Pt atom and reduce the number needed [7]. Single-atom catalysts (SACs), due to their unique coordination environment and maximum atomic utilization efficiency, have gained increasing attention [8]. In addition, controlling the morphology and sizes of Pt NPs offers an efficient alternative of highly efficient catalysts owing to the special crystal planes (dominant crystal planes, twins) and the arrangement of the atomic distribution on the surface [9].

The catalytic performance of Pt nanoparticle catalysts is closely related to their geometric surface properties and composition, so it is usually necessary to fine-tune the morphology, structure, and composition of metal nanocatalytic materials. There are currently many preparation methods for Pt, such as the electrochemical method [10,11,12,13,14], high-temperature organic synthesis method [15], colloid method [16], electrochemical deposition method [17], liquid-phase reduction method [18,19,20,21], microemulsion method [22], polyol method [23,24,25], and so on. The main differences between these methods are dispersants, reducing agents, reaction time or heat treatment methods, reaction atmosphere, etc. [26,27]. The above methods usually involve H_2_ or N_2_ atmospheres, excessive reaction time, or the use of potent reducing agents such as sodium borohydride (NaBH_4_) and other explosive reagents [28]. Additionally, most organic solvents, organic preservatives, and their vapors can pose potential hazards to the environment and human health. For example, oleic acid and polyamines, which are commonly used for morphology control in organic liquid-phase synthesis, can cause allergic reactions in human skin, while hydrazine hydrate (N_2_H_4_·H_2_O) and DMF can cause deformities and cancer in the human body, etc. [29,30]. Furthermore, the reaction time is often too long, usually about 3–6 h. In addition, the extensive use of organic solvents and surfactants with high toxicity leads to complications in the synthesis process, and the redundancy of reaction time, organic protective agents, and solvents that are adsorbed on the surface of the catalyst particles are not easily removed. The high price, non-recyclability and extensive use of organic solvents and preservatives significantly increase the cost of catalyst preparation and reaction time, limiting commercial applications and causing severe environmental pollution. Considering factors such as the preparation process, excessive reaction time, and environmental friendliness, the industrialization and commercial application prospects of these methods are limited. In previous work by our research group, Pt and PtCu concave nanoparticles with clean surfaces were successfully synthesized in a low-temperature molten salt system [31,32]. Therefore, exploring a new type of precious metal nanomaterial synthesis that is fast and is not based on organic solvents or organic protectants is particularly important for achieving low-cost, green synthesis and high-performance commercial applications of precious metal-based nanomaterials [33].

In recent years, many reports have shown that the microwave method can rapidly synthesize loaded platinum and platinum-based alloy nanoparticles with uniform particle size and high dispersion. For example, Tian et al. [34] synthesized platinum-based carbon nanotubes using intermittent microwave irradiation technology. Song et al. [35] prepared highly loaded Pt/C with a weight ratio of up to 50% using a pulsed microwave-assisted synthesis method. Chen et al. [36,37,38] prepared Pt and PtRu supported on carbon or carbon nanotubes with a uniform dispersion of 3 nm by microwave-heated polyol synthesis method. Compared with traditional heating methods, microwaves raise the reaction temperature more quickly and uniformly through special electromagnetic field effects and heating methods [39]. In addition, traditional synthesis methods typically take 3–6 h. Therefore, it is necessary to develop an efficient and time-saving method for the preparation of highly dispersed and highly loaded Pt/C catalysts. However, there are no reports from other research groups on the synthesis of noble metal nanomaterials in low-temperature molten salt using microwave ovens.

In this study, platinum nanostructures with concave structures were rapidly synthesized in a microwave oven and a mixed inorganic molten salt environment of potassium nitrate and lithium nitrate. Their morphology and size were continuously controlled in alkaline media and microwave irradiation, and the entire reaction time was around 15 min. Their electrochemical performance in formic acid was also investigated. Due to the absence of organic protective agents, the surface was very clean and showed excellent electrochemical catalytic activity and stability in formic acid catalysis. This work provides a universal environmentally friendly strategy for the rapid preparation of Pt and Pt-based alloy nanoparticles.

## 2. Results

### 2.1. Characterization of Pt Nanoparticles

**Figure 1:**
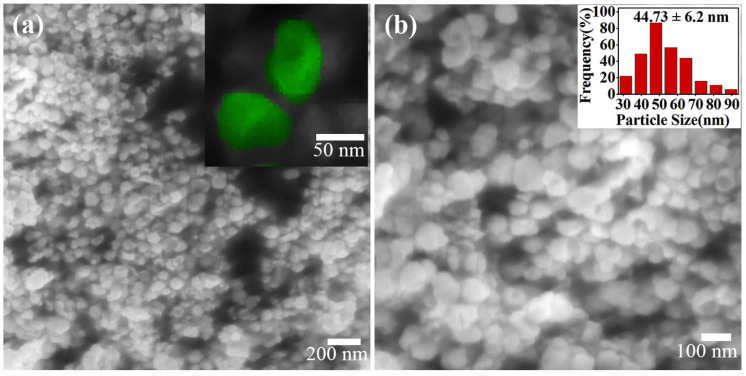
SEM (**a**) and magnified SEM (**b**) images of Pt concave NPs. The inset in (**a**) is the magnified SEM image of the Pt concave NPs, and the inset in (**b**) is the size distribution histogram.

Figure 1a,b show SEM and magnified SEM images of pure Pt concave NPs synthesized by the microwave method using a mixed molten salt of potassium nitrate and lithium nitrate as solvent. As can be seen in the inset of Figure 1a,b, the as-prepared NPs were relatively uniform in both size and shape, and almost all of the Pt NPs were irregularly concave in the center. The average size of the concave Pt NPs was about 44.73 ± 6.25 nm (inset in Figure 1b).

**Figure 2:**
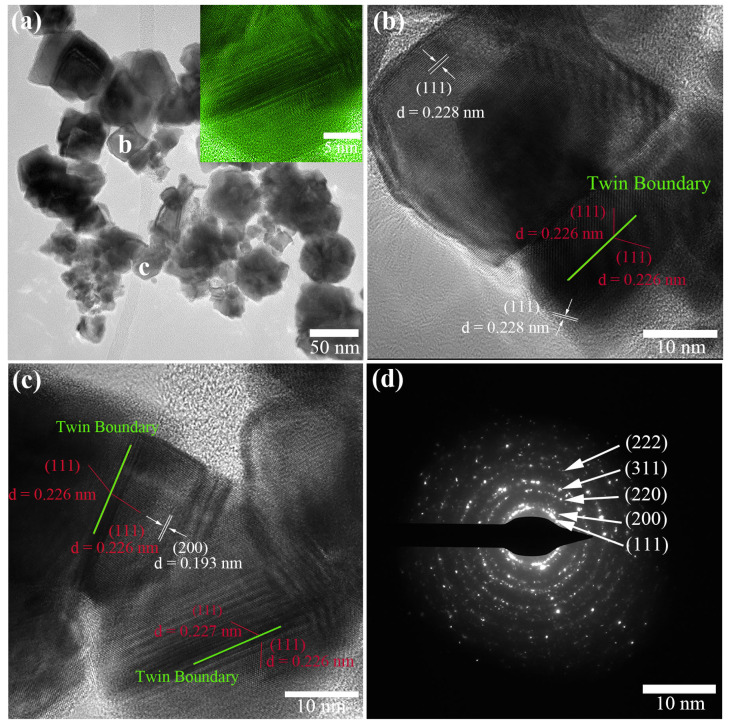
(**a**) TEM image of the Pt concave NPs. The inset is a typical individual nanoparticle with clear twin crystals. (**b**,**c**) HRTEM images of the Pt concave NPs labeled “b” and “c” in (**a**). (**d**) SAED pattern of the Pt concave NPs.

The structural details of the as-synthesized Pt concave NPs were characterized by TEM and HRTEM (Figure 2). It could be further confirmed that Pt with irregular concave surfaces at the center of the nanoparticles was successfully synthesized. As shown in Figure 2a, the Pt concave nanoparticles had a uniform size and a particle size of about 44 nm. The size was very close to the value measured by SEM images (illustrated in Figure 1b). It could also be clearly seen that there were regular strip-like structures on the surface of some nanoparticles. A close-up TEM image of a typical single twinned Pt nanoparticle (inset of Figure 2a) clearly presented a strip structure. A close-up TEM image of a typical single twinned Pt nanoparticle (Figure 2b,c) clearly presented a strip structure. High-resolution TEM (HRTEM) images further indicated that the striped bands were typically twinned nanostructures, with ordered atomic arrangements on one side that mirrored the symmetry of the other side’s atoms, segmented by twin boundaries (see Figure 2b,c). Figure 2b,c are HRTEM images of Pt concave NPs, which are marked “b” and “c” in Figure 2a. The lattice spacing of the Pt NPs is clearly revealed in the figure, and their lattice stripes show discontinuity and anisotropy, indicating that the Pt NPs had a polycrystalline structure [40,41]. The lattice stripes correspond to spacings of 0.228 and 0.193 nm, which agree well with the expected d-spacing of the (111) plane (0.226 nm) and (200) plane (0.196 nm) of bulk Pt crystals, respectively. Based on the above HRTEM image, it could be concluded that both crystal planes belong to (111) crystal plane, where (111) is the Miller index of twinned planes. The twin crystals grow periodically at a constant distance and exhibit long-range ordering, leading to the formation of symmetric superstructures in regular directions. The selected area electron diffraction (SAED) pattern obtained from a single particle showed an fcc structure (Figure 2d) consisting of several bright diffraction rings corresponding to the (111), (200), (220), (311), and (222) crystal planes, proving that the Pt/C concave nanoparticles were essentially polycrystalline structures, in agreement with the HRTEM results.

**Figure 3:**
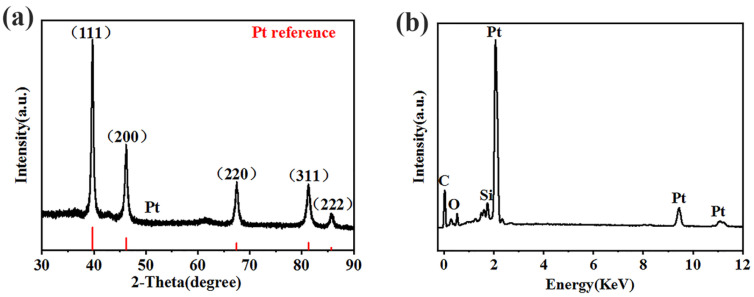
(**a**) XRD pattern of the Pt concave nanoparticles. (**b**) EDS spectrum of the Pt concave nanoparticles.

The XRD and EDS patterns of the Pt concave nanoparticles with reaction conditions of 300 W and 15 min are shown in Figure 3. In Figure 3a, it can be seen that the diffraction peaks of pure Pt are the (111), (200), (220), (311), and (222) faces of the face-centered cubic (fcc) structure. No other diffraction peaks were observed, indicating that the product was a pure phase. The observed diffraction angle of pure Pt was compared with the standard card (JCPDS database, 1999, PCPDFWIN version 2.02), indicating the formation of pure Pt NPs. From the EDX spectrum of the sample shown in Figure 3b, except for the C, O, and Si elements originating from the substrate (conductive silicone tape), only the signal peaks of platinum were observed, with no other impurity peaks present, indicating that the prepared product was pure platinum metal.

### 2.2. Preparation Conditions and Mechanism Analysis

In order to study the preparation conditions and mechanisms, the effects of different microwave powers and reaction times on the morphology and size of the product particles were investigated. Figure S1 shows the SEM images of the Pt concave nanoparticles under different microwave power preparation conditions. As shown in Figure S1, the as-prepared pure Pt NPs were relatively uniform in size and shape, with almost all NPs having an irregular concave shape at the center. The size distribution was measured and calculated based on the SEM images in Figure S1a–d. When the microwave power was below or above 300 W, the particle size distribution of the final product increased. For example, as shown in Figure S1a,d, the average particle size measured ranged from 44.73 nm for Pt concave NPs with a microwave power of 200 W to 58.15 nm for platinum nanoparticles with a microwave power of 1000 W. This was due to the fact that the increase in microwave power led to an increase in the temperature of the reaction. When the microwave power was 200 W, 300 W, 600 W, and 1000 W, the temperature of the salt bath of copper oxide was about 280 °C, 370 °C, 600 °C, and 840 °C, respectively. Figure S2 shows SEM images of Pt concave nanoparticles with different reaction times during the reaction process. It can be seen from the images that in the Pt concave nanoparticles, which formed at the reaction time of 5 s, the average particle size was particularly uneven. As the reaction time increased, the particle size became more uniform, and lots of nanoparticles began to have an irregular concave shape in the center. In Figure S2a–c, it can be seen that the average particle size of the self-prepared Pt NPs became more uniform with increasing reaction time.

Based on the above discussion and the formation mechanism of concave structured platinum nanoparticles with the same morphology synthesized by our research group using the molten salt method, the formation process of Pt nanoparticles is as follows. Firstly, Pt(NH_3_)_4_C_2_O_4_ powder was stirred and dispersed in KNO_3_-LiNO_3_ inorganic ionic liquid with a small amount of KOH at 180 °C. At this time, Pt(NH_3_)_4_C_2_O_4_ began to thermally decompose into Pt^0^ and release ammonia gas under the promotion of KOH (reaction Equation (1)). Due to the high viscosity of inorganic ionic liquid, ammonia gas will form bubbles and suspend in the system. As the number of Pt nuclei increases, they will grow into a metal shell through epitaxial growth at the gas–liquid interface, and further reactions will form a connected metal shell structure around the bubbles. Finally, the concave structured platinum nanoparticles were generated through the rupture of bubbles and the Ostwald ripening process [31,32].

Pt(NH_3_)_4_C_2_O_4_ + KOH → Pt + NH_3_ + K_2_CO_3_ + H_2_ (1)

The reduction method used for the precursor in this experiment was thermal decomposition. Generally, to thermally decompose tetraammine platinum oxalate, the temperature needs to be raised to above 200 °C. Below this temperature, a large amount of volatilization of tetraammine platinum oxalate will occur. To avoid the volatilization of the precursor, the reaction temperature must be lowered. If a thermal decomposition reaction occurs at a lower temperature, a thermal decomposition aid must be present. Considering that our research group previously used KOH-NaOH strong base inorganic ionic liquid, which can significantly reduce the decomposition temperature of precursors, we also chose to add a small amount of strong base as a thermal decomposition aid to promote the thermal decomposition of oxalic acid tetraammine platinum.

### 2.3. Electrochemical Performance Analysis

Figure S3a,b show the cyclic voltammetry curves of commercial Pt/C and the Pt concave NPs/C catalyst under reaction conditions of 300 W for 15 min in a 0.1 M HClO_4_ solution, respectively. Before conducting formic acid performance testing, residues adsorbed on the catalyst surface were removed and the active sites of the catalyst fully exposed [42]. The catalyst was first subjected to cyclic voltammetry testing. The Pt concave NPs/C catalysts exhibited typical characteristics of Pt catalysts in acidic electrolytes, with H^+^ adsorption/desorption peaks in the potential range of 0.02–0.3 V, smooth bilayer adsorption layers in the potential range of 0.3–0.6 V, and Pt oxide generation and reduction peaks in the potential range above 0.6 V. As shown in Table 1, the ECSA value of the catalyst was calculated by partial integration of the hydrogen adsorption region on the curve, where ECSA represents the number of active sites in the catalyst. The results showed that the ECSA values of the self-prepared Pt/C NPs and commercial Pt/C were 5.48 m^2^·g^−1^ and 45.90 m^2^·g^−1^ (Table 1), respectively. In comparison, the commercial Pt/C catalyst was found to have a higher ECSA value due to its smaller particles and better dispersion at the same Pt loading level [43]. A low ECSA value is a common issue for large catalysts. Many reports have indicated that catalytic activity was related to morphology [5], structure [1,44], and surface atoms [3,4,5,6], and a low ECSA may still possessed excellent catalytic performance [5,44,45].

**Figure 4:**
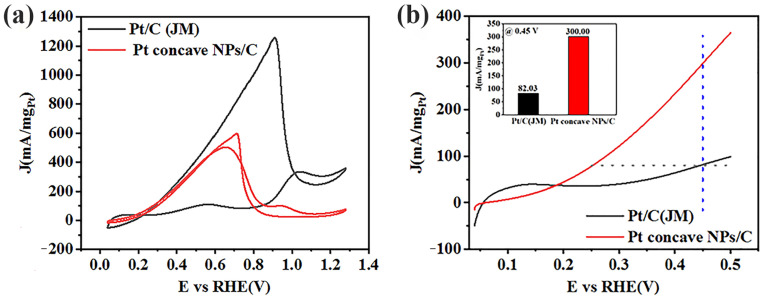
(**a**) Cyclic voltammograms (CVs) and (**b**) linear sweep voltammetry of 20% Pt/C (JM) and 300 W Pt concave NPs/C catalysts in 0.1 M HClO_4_ and 0.5 M HCOOH. Scan rate: 100 mV s^−1^. The inset shows the histogram of the mass current density at 0.45 V (vs. RHE) for Pt/C and 300 W Pt/C catalysts. Scan rate: 100 mV s^−1^.

The electrocatalytic properties of commercial Pt/C and Pt/C catalysts for formic acid oxidation were tested in a mixture of 0.1 M HClO_4_ and 0.5 M HCOOH, as shown in Figure 4a. Previous studies have shown that FAOR occurring on these catalysts mainly presents two distinctive pathways: the direct pathway proceeds via a dehydrogenation process, which is subsequently oxidized to CO_2_ (HCOOH → CO_2_ + 2H^+^ + 2e^−^); and the indirect pathway generates adsorbed CO species (COads) through a dehydration process, which can be oxidized to CO_2_ at higher potentials (HCOOH → COads + H_2_O→CO_2_ + 2H^+^ + 2e^−^) [46,47,48,49,50,51,52]. As shown in Figure 4, these catalysts exhibited two anodic oxidation peaks in the forward voltage scan during the electrooxidation of formic acid. The first anodic peak was the direct oxidation of formic acid to carbon dioxide, and its position in this region was not hindered by intermediate species (CO, etc.). At the higher potential, the second anodic oxidation peak being the indirect oxidation of formic acid, which is related to the oxidation of the adsorbed intermediate species (CO), which was oxidized and released free active sites, resulting in reduced catalytic activity. The oxidation of CO requires the formation of hydroxyl (OH) by water activation, where CO reacts with OH to form carboxyl (COOH) and then CO_2_ is formed by dehydrogenation [53]. Based on the experimental observation results and theoretical calculations presented in the aforementioned literature [47,48,49,50,51,52,53,54], as well as the observed results of the electrochemical curves for the catalytic oxidation of formic acid in our experiments, we hypothesize that the electrochemical oxidation of HCOOH on the self-made Pt concave NPs/C surface predominantly follows the direct pathway:

HCOOH + Pt → CO_2_* + H^+^ + e^−^

CO_2_* → CO_2_ (g)

with the overall reaction:

HCOOH + Pt → CO_2_ (g) + Pt + 2H^+^ + 2e^−^

On the basis of the ratio of the direct and indirect current peaks (j^d^)/(j^nd^) for the FAO reaction, the self-made Pt concave NPs/C and commercial Pt/C catalysts showed values of 4.97 and 0.33, respectively, indicating that the formic acid catalytic oxidation of the self-made Pt concave NPs/C catalyst was mainly direct, which reduces the poisoning effect of CO produced in the indirect pathway on the catalyst, thus enabling the catalyst to possess good catalytic durability [52,54]. In contrast, the formic acid catalytic oxidation of commercial Pt/C catalysts mainly follows the indirect oxidation pathway, and the catalysts are easily poisoned by CO.

In the reverse scan, the current reached a high anodic oxidation peak at about 0.7 V, which was attributed to the oxidation of a large amount of formic acid when CO did not poison the catalyst. However, when the potential reaches a more negative value, the catalyst surface is again poisoned by CO, resulting in a rapid drop in current [23]. The test results are shown in Figure 4a. The catalytic performance of the Pt/C catalyst for formic acid oxidation under reaction conditions of 300 W and 15 min was significantly better than the commercial Pt/C catalyst. The peak mass current densities of Pt/C NPs and commercial Pt/C were 502.00 mA·mg^−1^_Pt_ and 109.45 mA·mg^−1^_Pt_, respectively. The peak area current densities of Pt/C NPs and commercial Pt/C were 20.83 mA·cm^−2^_Pt_ and 0.22 mA·cm^−2^_Pt_, respectively (Figure S4 and Table 1). According to the calculations, the peak mass current density and peak area current density of the Pt/C NP catalysts were about 5 times and 20 times that of the commercial Pt/C catalysts, respectively. The peak current density of the direct oxidation peak was five times that of the indirect oxidation peak, and this indicates that the catalyst had particularly excellent anti-toxicity and formic acid electrochemical performance. However, the peak current density of the direct oxidation peak of commercial Pt/C was lower than that of the indirect oxidation peak, and this indicates that the self-made Pt/C catalyst had better electrochemical catalytic performance and anti-toxicity for formic acid than commercially available Pt/C catalysts. A comparison of Pt concave NPs/C with the same type of material is presented in Table 2.

The initial potential of the electrochemical catalytic oxidation of formic acid was an important parameter used to evaluate the electrochemical catalytic activity at low voltage. As shown in Figure 4b, the initial potential of the Pt concave NPs/C catalyst was more negative than that of the Pt/C (JM) catalyst. In addition, at the potential corresponding to the given oxidation current density, the potential of the Pt concave NPs/C catalyst was still the smallest (dashed gray line in Figure 4b). At a given voltage of 0.45 V, the mass current density of the Pt concave NPs/C catalyst (300.00 mA·mg^−1^_Pt_) was approximately 3.7 times that of the Pt/C (JM) catalyst (82.03 mA·mg^−1^_Pt_) (blue dashed line and illustration in Figure 4b). To verify the reliability and reproducibility of the experimental results, the electrochemical catalytic oxidation performance of formic acid was tested for seven different batches of Pt concave NPs/C catalyst samples. The results are shown in Figure S5 and Table S1. The mean values and standard error of the mass current density at 0.45 V for the commercial Pt/C catalyst and the self-made Pt concave NPs/C catalyst were 78.09 ± 4.89 and 288.22 ± 58.73 mA·mg^−1^_Pt_, respectively. This indicated that the Pt concave NPs/C catalysts had superior electrochemical catalytic performance for formic acid compared to the commercial Pt/C catalyst.

The prepared concave Pt nanoparticles exhibited excellent formic acid catalytic oxidation properties despite their large size, which could be attributed to the following reasons. Firstly, the catalyst prepared by the microwave-assisted low-temperature molten salt method has a very clean surface without any organic coating, requiring no cleaning before use. The platinum atoms are completely exposed, allowing them to exhibit a much enhanced electrocatalytic oxidation performance, which has been verified in our previous work [31,32]. Secondly, the twin boundaries on the catalyst surface play a crucial role. Twin crystals with highly ordered atomic arrangements are efficient and dependent planar defects in NPs for further enhancing carrier separation and transfer efficiency due to their induced high electrical conductivity and strong built-in electric field, which is well supported by DFT calculations and a series of experimental evidence [9]. Lastly, the main pathway for our self-made catalyst oxidation of formic acid was the direct pathway, which avoids CO poisoning and enhances durability [54].

**Figure 5:**
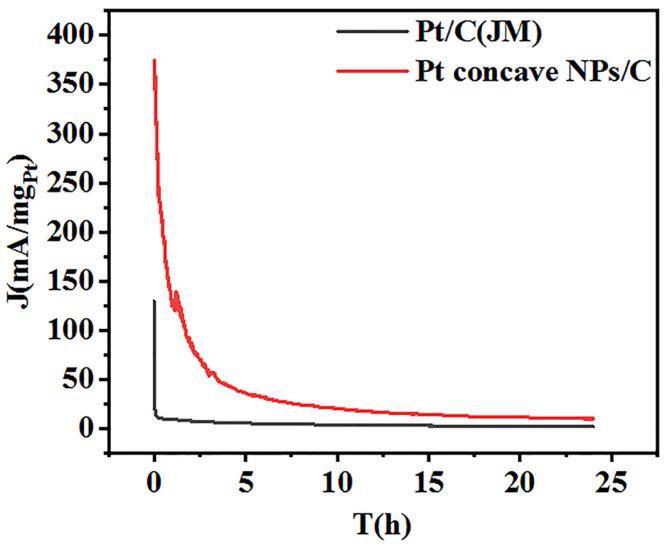
Current–time curves of Pt/C (JM) and Pt concave NPs/C catalysts for the electrooxidation of formic acid at 0.45 V in 0.1 M HClO_4_ and 0.5 M HCOOH.

The stability of Pt concave NPs/C and commercial Pt/C catalysts were tested using chronoamperometry, with measurements taken at a constant potential of 0.45 V (vs. RHE) for 24 h and in a 0.1 M HClO_4_ and 0.5 M HCOOH electrolyte. As shown in Figure 5, the mass current density of the Pt concave NPs/C and commercial Pt/C catalysts decreased rapidly at the beginning of the reaction, and the sharp decline in catalyst performance can be explained by the decrease in formic acid concentration gradient on the catalyst surface and catalyst poisoning. The Pt concave NPs/C catalyst exhibited an initial mass-specific activity value of 300.00 mA·mg^−1^_Pt_ and a final mass-specific activity value of 10.91 mA·mg^−1^_Pt_ after 24 h. In comparison, the Pt/C catalyst produced an initial mass-specific activity value of 82.03 mA·mg^−1^_Pt_ and a final mass-specific activity value of 2.35 mA·mg^−1^_Pt_. The result demonstrates that the electrochemical stability of the Pt concave NPs/C catalyst towards formic acid oxidation was much higher than that of the Pt/C (JM) catalysts [57]. This result, combined with CV measurements, further confirmed that the Pt concave NPs/C catalyst was superior to the catalytic activity, antitoxicity, and stability of Pt/C (JM) for the electro-oxidation of HCOOH.

## 3. Experimental Sections

### 3.1. Preparation of Pt Nanoparticles

**Figure 6:**
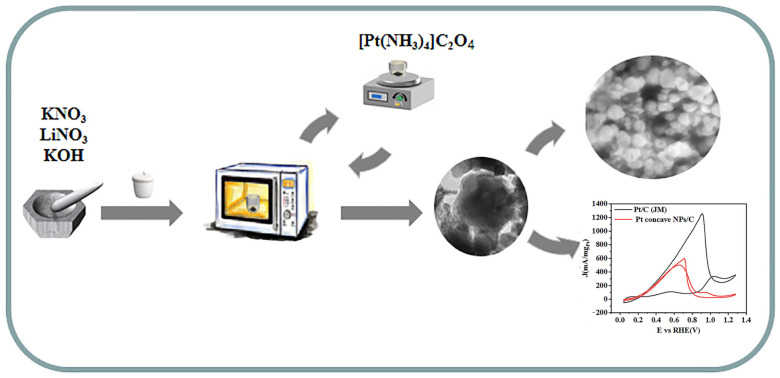
Schematic diagram of the preparation process of Pt nanoparticles.

The Pt electrocatalysts were easily and rapidly prepared by a novel microwave method. The main steps of this synthesis process are as follows (Figure 6): 0.66 g of KNO_3_ (Tianjin Hengxing Chemical Reagent (Tianjin, China), AR, 99.0%), 0.34 g of LiNO_3_ (Aladdin Reagent (Shanghai) Co., Ltd. (Shanghai, China), CR, 98.0%), and 0.0224 g of KOH (Tianjin Hengxing Chemical Reagent, (Tianjin, China), AR, 85.0%) were mixed and uniformly ground in an agate mortar. The mixture was placed in a 30 mL crucible, then the entire crucible was placed in a 100 mL crucible containing CuO and subjected to microwave reaction at 200 W for 15 min. After 15 min, 5 mg of Pt(NH_3_)_4_C_2_O_4_ (Aladdin Reagent (Shanghai) Co., Ltd. (Shanghai, China), AR, 99.0%) was added to the mixture of KNO_3_ and LiNO_3_ inorganic ions in the liquid, stirred with magnetic force for 1 min, and finally the smaller crucible was transferred to the 100 mL crucible for microwave reaction at 300 W for 15 min. After the reaction, the product was sonicated continuously for 15 min, cleaned twice with distilled water, washed once with anhydrous ethanol, and then placed in a bake-out oven at 45 °C for 12 h.

### 3.2. Preparation of 20% Pt/C Catalyst

The Pt/C catalyst was prepared by loading as-prepared Pt concave NPs on carbon black (Vulcan XC-72, Cabot Corporation, Boston, MA, USA) (Figure S6). In a normal procedure, 1 mg of Pt NPs and 2 mL of C_2_H_5_OH (Tianjin Hengxing Chemical Reagent (Tianjin, China), AR) were added to a sample bottle and sonicated for 15 min. Carbon black (4 mg) was dispersed in 20 mL of C_2_H_5_OH and sonicated for 0.5 h before the addition of Pt NPs. The amount of Pt NPs was fixed at 20 wt% of the final product. The mixtures were stirred for 24 h before the final products were collected by centrifugation at 5000 rpm for 3 min. The precipitate was dried in an oven at 45 °C for 12 h.

### 3.3. Characterization of Pt Nanoparticles

The surface morphology and microscopic internal structure of the samples were determined by scanning electron microscopy (SEM) and transmission electron microscopy (TEM). The atomic compositions of the Pt NPs were analyzed by energy-dispersive X-ray spectroscopy (EDX). Powder X-ray diffraction (XRD) was performed on a Bruker D8 advanced X-ray diffractometer.

### 3.4. Electrochemical Testing

Electrochemical measurements were performed on three-electrode system electrolytic cells and an electrochemical workstation (Chenhua CHI 660E, Shanghai Chenhua Instrument Co., Ltd., Shanghai, China) using typical cyclic voltammetry (CV) techniques. An Ag/AgCl (3 M KCl) electrode was used as the reference, a rotating disk electrode (RDE) as the working electrode, and a 1 cm × 1 cm platinum mesh (Pt purity: 99.9%) as the counter electrode. All potentials in this paper refer to reversible hydrogen electrodes (RHEs). The electrolyte used for all measurements was 0.1 M perchloric acid (HClO_4_) diluted from a stock solution (Tianjin Xinyuan Chemical Reagent (Tianjin, China), GR, 70.0–72.0%) with distilled water. To prepare the working electrode, deionized water, isopropanol, and 5% Nafion solution were evenly mixed in a volume ratio of 4:1:0.025 and sonicated for 15 min. Pt concave NPs/C catalyst (1 mg) was sonicated for 15 min in 1 mL of the mixed solution. A portion (40 μL) of the suspension was added to the glassy carbon electrode and dried at room temperature, with a final Pt loading of 8 μg. Electrochemical surface area (ECSA) measurements were determined by integrating hydrogen adsorption from cyclic voltammetry (CV) recorded at room temperature in an argon-saturated 0.1 M HClO_4_ solution. The cyclic voltammetry (CV) measurements were performed in 0.1 M HClO_4_ solution, which was purged with nitrogen for 30 min at a scan rate of 50 mV·s^−1^ and a voltage range of 0.02 to 1.1 volts (vs. RHE). The catalytic oxidation of formic acid experiments was performed in 0.1 M HClO_4_ and 0.5 M HCOOH (Aladdin Reagent (Shanghai) Co., Ltd. (Shanghai, China), HPLC, 99.0%) solution at a scan rate of 100 mV·s^−1^ and a voltage range of 0.04 to 1.28 volts (vs. RHE) for CV measurement. To obtain a stable voltammetric curve, it was generally necessary to perform 50 cycles of electrochemical pretreatment, which can play a role in electrochemical impurity removal and activation. For comparison, Pt/C (JM, 20 wt%) was used as a reference catalyst, and the same procedure as described above was used to perform the electrochemical measurement. In all electrochemical tests, the electrolyte solution temperature was maintained at 25 °C.

Electrochemical active surface area (ECSA) refers to the effective area of an electrochemical reaction, which can be used to determine the number of active sites in a catalyst. The ECSA value of the catalyst can be obtained by integrating the hydrogen adsorption region (0.05–0.40 V vs. RHE, a reversible hydrogen electrode) of the cyclic voltammetry curve measured in 0.1 M HClO_4_ solution. By integrating the hydrogen adsorption region, the ECSA value of the catalyst can be obtained, which can be used to determine the number of active sites exposed by the catalyst [32]. The specific calculation formula is as follows:

$$ECSA=\frac{O_{de}}{m\times C}$$

where $O_{de}$ is the total adsorption capacity, m is the mass of Pt loaded on the electrode, and C is the adsorption capacity per unit Pt surface area. In this study, a 0.1 M HClO_4_ solution saturated with N_2_ was used as the electrolyte. Cyclic voltammetry tests were performed at a sweep rate of 50 mV·s^−1^ at a voltage of 0.02–1.10 V (vs. RHE), and the value of ECSA was calculated.

## 4. Conclusions

In conclusion, uniform Pt concave nanoparticles can be rapidly and successfully synthesized in low-temperature molten salt by a microwave method without using any organic solvents, capping agents, or structure-directing agents. Due to microwave irradiation, the obtained product had well-controlled size and size distribution, and the catalyst exhibited excellent antitoxicity, stability, and catalytic activity in the direct oxidation of formic acid. It achieved peak mass current density values up to 500 mA·mg^−^^1^_Pt_ and peak area current density values up to 20.83 mA·cm^−^^2^_Pt_. In addition, the mass-specific activity value of the Pt concave NPs/C catalyst (10.91 mA·mg^−^^1^_Pt_) was approximately 4.5 times that of Pt/C (JM) (2.35 mA·mg^−^^1^_Pt_) after a 24 h chronoamperometry test. The main pathway for the electrocatalytic oxidation of formic acid using the self-made Pt concave NPs/C catalyst was the direct oxidation pathway, which reduced the poisoning effect of CO generated in the indirect pathway on the catalyst, and thus the Pt concave nanoparticles exhibited much higher catalytic activity and stability for formic acid electrooxidation than commercially used Pt/C electrocatalysts. The microwave-assisted low-temperature molten salt method can rapidly penetrate the material and directly transfer the energy to the interior of the molecules, which makes the heating more efficient. This can be extended to the preparation of platinum-based alloys as well as other catalysts. In the next step, the preparation of smaller nanoparticles and single-atom catalysts, as well as directly loading catalysts onto activated carbon or other carriers with better performance, will require focused research.

## Figures and Tables

**Table 1:** ECSA, mass current densities, and initial potential of Pt concave NPs/C and Pt/C (JM).

Catalyst	ECSA	Peak Mass Current Densities	Peak Area Current Density	Mass Current Density at 0.45 V	Initial Potential Value
Pt/C	5.48 m^2^/g	502.00 mA/mg_Pt_	20.83 mA/cm^2^_Pt_	300.00 mA/mg_Pt_	0.202 V
Pt/C (JM)	45.90 m^2^/g	109.45 mA/mg_Pt_	0.22 mA/cm^2^_Pt_	82.03 mA/mg_Pt_	0.305 V

**Table 2:** Comparison of formic acid electrochemical properties of Pt NPs/C with other material.

Material	Method	Peak Mass Current Density	Peak Area Current Density	Electrolyte	Ref.
Pt/C	Co-reduction	150 mA·mg^−1^_Pt_	-	0.5 M H_2_SO_4_ + 0.5 M CHOOH	[21]
Pt/C	-	49.43 mA·mg^−1^_Pt_	-	0.5 M H_2_SO_4_ + 0.5 M CHOOH	[46]
Pt/C	Ultrasound-assisted	95.00 mA·mg^−1^_Pt_	-	0.5 M H_2_SO_4_ + 1 M CHOOH	[55]
PtCu/C	Molten salt	-	2.15 mA·cm^−2^_Pt_	0.1 M HClO_4_ + 0.5 M CHOOH	[32]
PtCu spherical	Polyol	-	0.95 mA·cm^−2^_Pt_	0.1 M HClO_4_ + 0.5 M CHOOH	[23]
PtCu cubic	Colloidal	-	1.12 mA·cm^−2^_Pt_	0.1 M HClO_4_ + 0.5 M CHOOH	[56]
Pt spherical	Polyol	-	0.45 mA·cm^−2^_Pt_	0.1 M HClO_4_ + 0.5 M CHOOH	[23]
Pt NPs/C	Microwave	502.00 mA·mg^−1^_Pt_	20.83 mA·cm^−2^_Pt_	0.1 M HClO_4_ + 0.5 M CHOOH	This work

## Data Availability

The original contributions presented in the study are included in the article/Supplementary Materials. Further inquiries can be directed to the corresponding authors.

## Supplementary Materials

The following supporting information can be downloaded at https://www.mdpi.com/article/10.3390/molecules29215128/s1. Figure S1: SEM images of products prepared with different microwave reaction powers; Figure S2: SEM images of products prepared with different microwave reaction time; Figure S3: Cyclic voltammogram (CV) curves of (a) 20 % Pt/C(JM) and (b) 300 W Pt concave NPs/C in 0.1 M HClO_4_; Figure S4: Cyclic voltammograms (CVs) of 20% Pt/C(JM) and 300W Pt concave NPs/C catalysts in 0.1 M HClO_4_ and 0.5 M HCOOH; Figure S5: Cycle voltammograms (CV) of seven different batches of (a) Pt/C (JM) and (b) self-made Pt concave NPs/C in 0.1 M HClO_4_ and 0.5 M HCOOH, respectively; Figure S6: TEM image of the Pt concave loaded on the vulcan carbon; Table S1: Electrochemical catalytic performance test results of seven different batches of Pt/C(JM) and Pt concave NPs/C.
