# Supplementary material for: Rapid Preparation of Platinum Catalyst in Low-Temperature Molten Salt Using Microwave Method for Formic Acid Catalytic Oxidation Reaction
Source: Molecules. 2024 Oct 30;29(21):5128. doi: 10.3390/molecules29215128 (PMC11548013; doi:10.3390/molecules29215128)
Supplement: Supplementary file 1 [file molecules-29-05128-s001.zip › molecules-3211461-supplementary.pdf]

Rapid preparation of platinum catalyst in low-temperature molten salt using microwave method for formic acid catalytic oxidation reaction

Haidong Zhao[1], Xiaoyan Hu[1], Hongbiao Ling[1], Ji Li[1], Weixu Wang[1], Jingtao Guo[1], Rui Liu[1], Chao Lv[2], Zhen Lu[1,*], Yong Guo[1,*]

1 School of Chemistry and Chemical Engineering, Shanxi Datong University, Datong, 037009, China;
2 School of Coal Engineering, Shanxi Datong University, Datong, 037009, China;
* Correspondence: luzhen0313@aliyun.com; ybsy_guo@163.com (Y. G.);

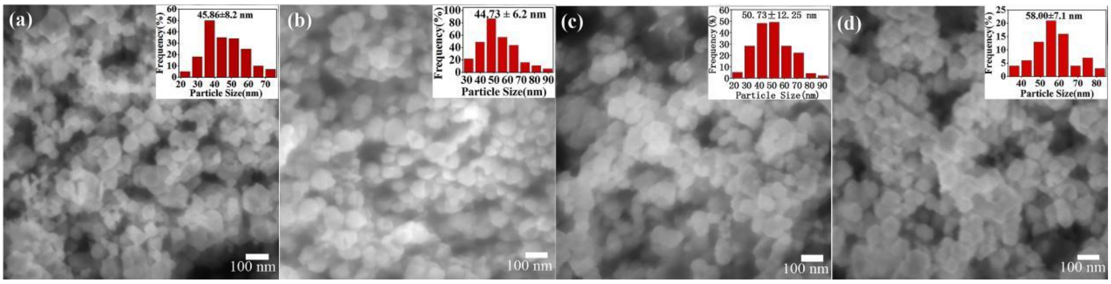

Figure S1 SEM images of products prepared with different microwave reaction powers. (a) 200 W, 15 minutes; (b) 300 W, 15 minutes; (c) 600 W, 15 minutes; (d) 1000 W, 15 minutes. The inset images in (a), (b), (c) and (d) are the size distribution histograms for the Pt nanoparticles.

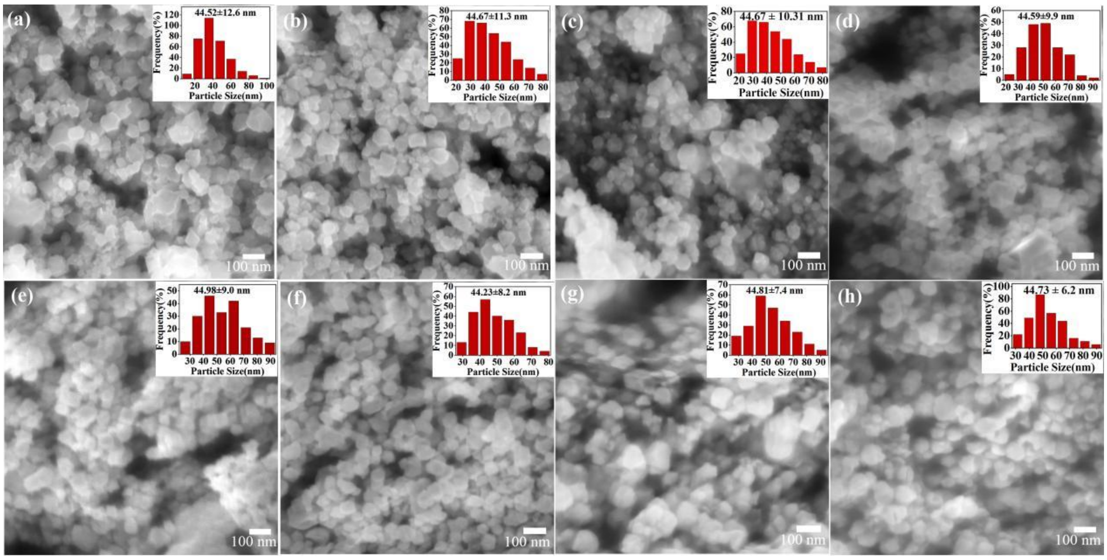

Figure S2 SEM images of products prepared with different microwave reaction time. (a) 300 W, 5 S; (b) 300 W, 15 S; (c) 300 W, 30 S; (d) 300 W, 1 min; (e) 300 W, 3 min; (f) 300W, 6 min; (g) 300 W, 10 min; (h) 300 W, 15 min. The inset images in (a), (b), (c), (d), (e), (f), (g) and (h) are the size distribution histograms for the Pt nanoparticles.

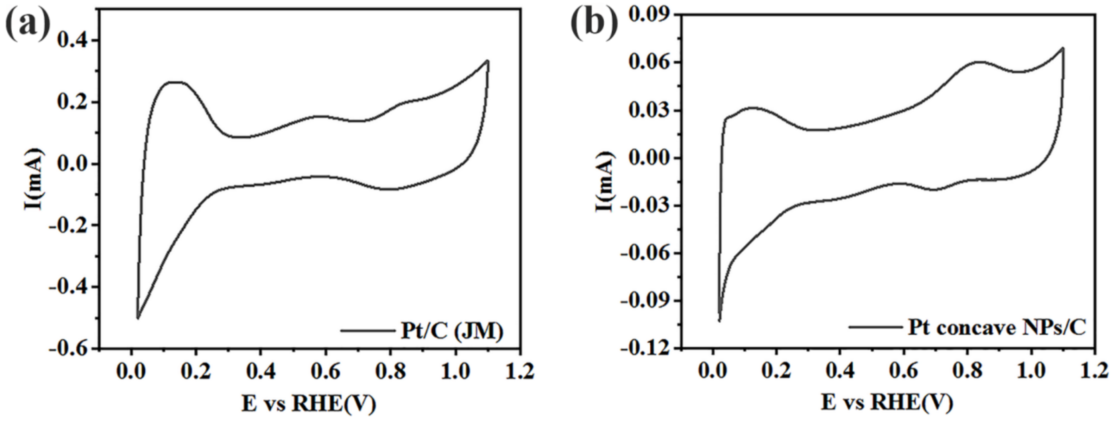

Figure S3 Cyclic voltammogram (CV) curves of (a) 20 % Pt/C(JM) and (b) 300 W Pt concave NPs/C in 0.1 M $HClO_4$, scan rate: 50 mV s^{-1}.

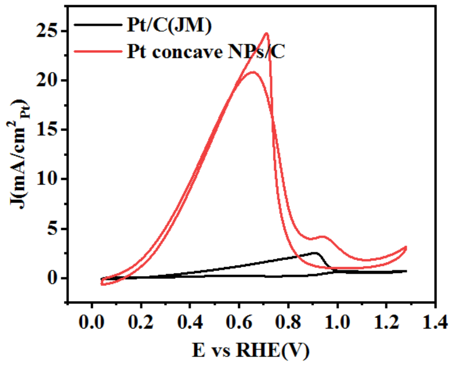

Figure S4 Cyclic voltammograms (CVs) of 20% Pt/C(JM) and 300W Pt concave NPs/C catalysts in 0.1 M $HClO_4$ and 0.5 M HCOOH. Scan rate: 100 mV s-1.

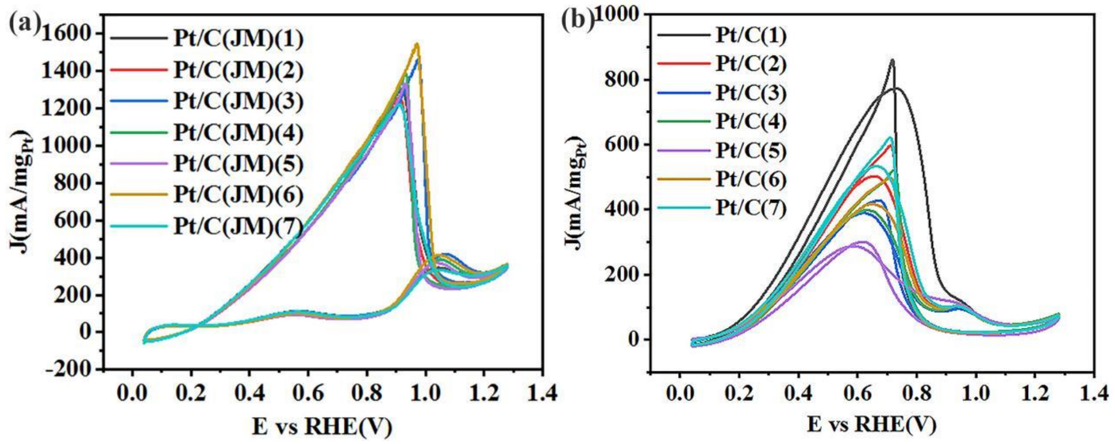

Figure S5 Cycle voltammograms (CV) of seven different batches of (a) Pt/C (JM) and (b) self-made Pt concave NPs/C in 0.1 M $HClO_4$ and 0.5 M HCOOH, respectively. Scanning rate: 100 mV s^{-1}.

Table. S1 Electrochemical catalytic performance test results of seven different batches of Pt/C(JM) and Pt concave NPs/C

Catalyst	ECSA (m^2/g)	Peak mass current densities (mA/mg_{Pt})	Peak area current density (mA/cm^2_{Pt})	Mass current density at 0.45V (mA/mg_{Pt})	Mean value with error bars	Initial potential value (V)
Pt/C(JM) (1)	47.60	112.48	0.24	82.93		0.309
Pt/C(JM) (2)	45.90	109.45	0.22	82.03		0.305
Pt/C(JM) (3)	50.00	111.55	0.24	79.78	78.09±4.8	0.312
Pt/C(JM) (4)	45.00	101.96	0.23	74.90	9	0.291
Pt/C(JM) (5)	44.10	93.31	0.22	70.67	mA/mg_{Pt}	0.305
Pt/C(JM) (6)	45.40	100.28	0.23	73.88		0.301
Pt/C(JM) (7)	44.20	112.14	0.26	82.41		0.312
Pt concave/C (1)	5.63	773.13	27.03	403.38		0.186
Pt concave/C (2)	5.48	502.00	20.83	300.00		0.202
Pt concave/C (3)	2.42	389.88	7.09	266.63	288.22±58	0.249
Pt concave/C (4)	4.89	397.75	8.16	257.25	.73	0.253
Pt concave/C (5)	2.44	287.25	11.49	219.63	mA/mg_{Pt}	0.230
Pt concave/C (6)	3.19	416.50	12.82	261.38		0.233
Pt concave/C (7)	5.84	532.89	25.02	309.25		0.203

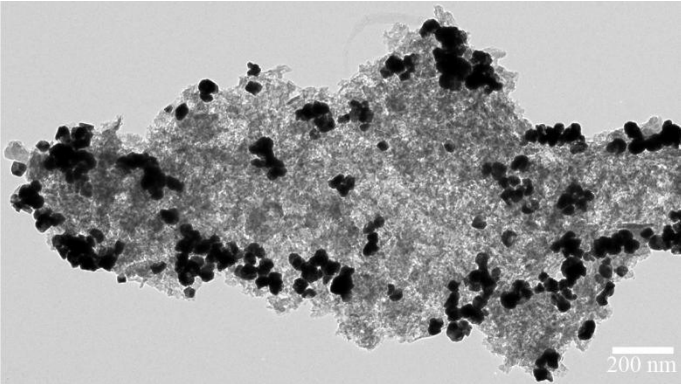

Figure S6 TEM image of the Pt concave loaded on the vulcan carbon
